# Evaluation of the heavy metals (mercury, lead, and cadmium) contamination of sardine (*Sardina pilchardus*) and swordfish (*Xiphias gladius*) fished in three Algerian coasts

**DOI:** 10.14202/vetworld.2019.7-11

**Published:** 2019-01-02

**Authors:** Fetta Mehouel, Leila Bouayad, Abdel Hamid Hammoudi, Ouarda Ayadi, Fifi Regad

**Affiliations:** 1 Laboratory of Food Hygiene and Quality Insurance System, High National Veterinary School, Algiers, Algeria; 2 Laboratory of Microbiology, Institute of Veterinary Sciences, Tiaret, Algeria; 3 Laboratory of Parasitology, Institute of Veterinary Sciences El Khroub, University of Frères Mentouri, Constantine 1, Algeria; 4 Laboratory of the Water and Sanitation Society, Algiers, Algeria

**Keywords:** atomic emission spectroscopy, fish, hazard, heavy metal, sardine, swordfish

## Abstract

**Aim:**

This study aimed to evaluate mercury (Hg), cadmium (Cd), and lead (Pb) levels in 70 samples of sardine (*Sardina pilchardus*) and 30 samples of swordfish (*Xiphias gladius*) fished in the Algerian coasts.

**Materials and Methods:**

After the mineralization of the fish samples through the pressure digestion, the analyses were carried out by inductively coupled plasma atomic emission spectroscopy.

**Results:**

Mean concentrations of Hg, Cd, and Pb in sardine were 0.62, 0.55, and 2.13 mg/kg wet weight, respectively, while in swordfish, the concentrations were 0.56, 0.57, and 3.9 mg/kg wet weight, respectively. These results exceeded the Algerian and European legislation threshold values, whereas Hg’s concentration in swordfish remained close to and did not exceed the recommended thresholds (0.56 mg/kg wet weight).

**Conclusion:**

This fish may represent a hazard for consumers in Algeria. Systematic and periodic controls of heavy metals in fish are recommended, and risk assessment is needed to protect the consumer.

## Introduction

Fish have been acknowledged as an integral component of a well-balanced diet, providing a healthy source of energy, high-quality proteins, vitamins, and a wide range of other important nutrients [1]. Moreover, fish are a significant source of omega-3 polyunsaturated fatty acids whose benefits in lowering the risk of coronary heart disease and on normal neurodevelopment in children have been widely documented [1]. Heavy metals discharged into the aquatic environment from different natural and human activities sources, including industrial or domestic wastewater, application of pesticides and inorganic fertilizers, leaching from landfills, shipping and harbor activities, atmospheric deposits, and geological weathering of the earth crust [2], can damage fish species diversity as well as ecosystems, due to their long persistence, ability to bioaccumulation, and toxicity [3].

Fish accumulate heavy metals from food, water, and sediments. The content of toxic heavy metals in fish can impair their beneficial nutritional attributes [4]. Among a wide range of toxic substances that contaminate fish and seafood, three heavy metals; cadmium (Cd), lead (Pb), and mercury (Hg) are the only heavy metals included in the European Union regulations for hazardous metals [5]. Heavy metals can interfere with biological systems and have inappropriate interactions with different intracellular structures. They are highly toxic to marine organisms and human, even at very low concentrations due to bioaccumulation. Hence, their presence even at a trace level in fish might constitute a serious threat to the health of consumers. Chronic exposure to Hg and Hg compounds is harmful to human health, especially to the fetuses and children at early stages of development [6]. This metal can cause most damage and dysfunction of the central nervous system (CNS). Chronic exposure to Pb is deleterious for the hematological system, the CNS, and the renal system. Cd long-term exposure impairs kidney’s normal functioning [7]. The pollution of marine ecosystems is a worldwide problem, and the situation is aggravated by the ability of these ecosystems to concentrate and accumulate some metals within the food chains [7]. Fish can concentrate very high levels of these contaminants, sometimes exceeding authorized limits. This bioaccumulation is closely related to the place of some fish species at the top of the aquatic food chain [6]. For this reason, determination of the chemical quality of aquatic organisms, particularly their heavy metals contents, is important for human health [8]. In Algeria, sardine (*Sardina pilchardus*) is a commercially important species, because it is the most consumed fish [9].

In this study, we evaluated heavy metals conta mination levels (Hg, Pb, and Cd) in sardine (*S. pilchardus*) and swordfish (*Xiphias gladius*) fished in Algerian (Centre and east and west Algerian) coasts, and then, we appreciate the distribution of the concentrations of these contaminants in the three studied areas.

## Materials and Methods

### Ethical approval

Ethical approval is not applicable to this study as fish samples used for the analysis of heavy metals were collected from fisheries and market stalls in Algeria.

### Sampling and sample preparation

**Figure-1:**
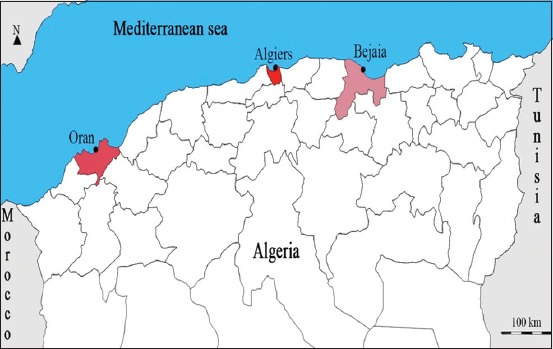
Geographical location of the Algerian area where the fish’s samples were collected.

A total number of 100 fish samples were collected between May and December 2015. It consists of 30 samples of swordfish (*X. gladius*) bought from the fish markets stalls of Algiers, and a total number of 70 sardine’s samples (*S. pilchardus*) which were collected from the port fisheries of three Algerian coast major ports (Figure-1); 30 from the coast of Algiers (North Centre), 25 from the coast of Bejaia (North-East), and 15 from the coast of Oran (North-West). These coasts are exposed to heavy marine pollution due to their proximity to big cities [10]. Sampling was performed according to the European Regulation [5]. Flesh samples (300 mg) were taken from each fish sample to obtain 200 g of the recommended flesh matrix after the removal of inedible parts [11]. The samples were packed in polyethylene bags blanks and transported to the laboratory in ice containers.

Sample preparation was performed to reduce the risk of any exogenous contamination; samples were quickly prepared after their arrival in the laboratory in compliance with two European standards: EN 13804 (2013) and EN 13805 (2002). Fishes were rinsed with tap water then distilled water.

Before each set of analyses, a blank test was carried out to confirm the absence of any contaminant in the apparatus and to determine the limits of detection and quantification of the measuring apparatus. The test blank was conducted in the absence of matrix (fish flesh) with the same quantities of reagents and subjected to mineralization at the same time as the samples. 10 successive measurements of the blank were performed to eliminate the samples whose concentrations are lower than the detection limits. The detection limits are equal to 3 times the standard deviation of the blanks.

### Mineralization of samples

Samples mineralization was done according to European Standard EN 13805 (2002). Briefly, 2 g of each tested sample was put in Teflon bombs, 3 mL of nitric acid and 0.5 mL of hydrogen peroxide were added to avoid the deposition of the sample on the walls of the vessel and to obtain a homogeneous mixture with the acid. The digestion vessel put into a pressure container was placed in a heat source (muffle furnace) for 3 h at 170°C. After cooling, the digestion solution was placed in an ultrasound bath for 10 min at 25°C to degas the solution. The latter was then transferred into 10 mL test tubes and supplemented with distilled water before storage at 4°C.

### Analysis and calibration

Before each series of measurements, commercialized calibration solutions were prepared (6 for Pb and Cd and 5 for Hg); then, calibration curves were established. For Pb and Cd, a standard (PerkinElmer Pure reference N9300281) was used at a concentration of 100 mg/L for Hg; a standard (AccuStandard reference AA34N-5) was used at a concentration of 1000 mg/L.

The determination of heavy metals concentration was performed using inductively coupled plasma atomic emission spectrometer PerkinElmer-Optima 800. The calibration of the device was performed by the embodiment of 10 blank measurements.

### Quality control of the analysis

Analytical performances were verified by processing Certified Reference Materials:

For Pb, As, and Cd, we used the cereals and derivatives BIPEA with internal reference number (15350395) and a known concentrations of these metals (As=0.332 mg/kg w.w), (Cd=0.406 mg/kg w.w), and (Pb=0.851 mg/kg w.w). The results were in good agreement with the certified values located in these intervals: 0.5552-0.4085 for As, 0.3046-0.5075 for Cd, and 0.6128-0.10892 for Pb.

For Hg, we used the canned fish (Fapas) with an internal reference number (12130869) and the tissue of lyophilized mussels ERM-CE278k with known concentrations of Hg (0.359 and 0.071mg/kg w.w), respectively. The results were in good agreement with the certified values 0.404-0.674 mg/kg w.w and 0.053-0.089 mg/kg w.w for both reference materials, respectively.

### Statistical analysis

The descriptive statistics (mean, standard deviation, minimum values, and maximum values) were estimated using Microsoft Excel^®^ (2007) software.

R version 3.0.2. was used for the analysis of the variance to compare the concentrations according to the fishing zones of sardine. In case of significant differences, the Newman-Keuls test was used to establish the homogeneous groups. The non-parametric Mann–Whitney test was used to compare the differences in the metal content in the two fish species. The threshold value for all the statistical tests was 5%.

## Results

The detection limits of Cd, Pb, and Hg were 1.03×10−^2^, 4.4×10−3, and 1.8×10−3 mg^/^kg, respectively (Table-1). The determination of the different heavy metals concentrations in the flesh of the two species showed a dominant Pb contamination compared to the two other metals with a higher concentration in swordfish than in the sardine (3.90±2.79 and 2.13±1.12), respectively (Table-2). Cd concentration is also higher in swordfish (Table-2). The Mann–Whitney test showed a significant difference in the concentrations of these metals between the two species, with p<5%. Hg recorded almost similar concentrations between the two species, the Mann–Whitney test showed no significant difference, with p>5%.

**Table-1:** Limits of detection and quantification of mercury, cadmium, and lead (mg/kg).

Heavy metals	Cadmium	Lead	Mercury
Limit of detection (3×standard deviation)	0.0103	0.0044	0.0018
Limit of quantification (3×limit of detection)	0.0309	0.0378	0.0054

**Table-2:** Variations in cadmium, lead, and mercury concentrations in sardine and swordfish flesh (mg/kg fresh weight).

Fish species	n	Mean±SE (Max-Min)		

		Cadmium	Lead	Mercury
Sardine	70	0.55±0.44 (UD-2.11)	2.13±1.12 (0.51-7.53)	0.62±0.16 (0.15-0.89)
Swordfish	30	0.57±1.34 (0.018-7.52)	3.90±2.79 (0.02-11.40)	0.56±0.15 (0.27-0.88)

n=Number of samples, SE=Standard deviation, Min=Minimum, Max=Maximum, UD=Concentration under the limit of detection

**Figure-2:**
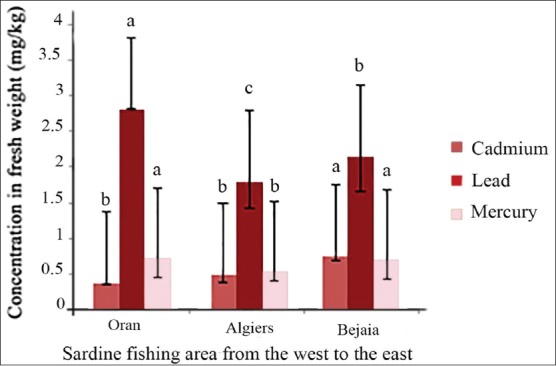
Variations of cadmium, lead, and mercury concentrations in sardine flesh according to the fishing area (mg/kg fresh weight). (a, b, c) Homogeneity between fishing areas for each metal is designed by the same letter.

The Newman-Keuls test showed homogeneity between the mean Cd concentrations for sardine fished in center and west, for Hg concentration in west and east, whereas for Pb, this test showed no homogeneity for the three fishing areas (Figure-2).

## Discussion

The detection limits of Cd, Pb, and Hg were higher than those reported by Olmedo *et al*. [1] and Zaza *et al*. [12]. These variations are explained by differences in analytical conditions that include different matrices, analytical methodology, and equipment. Heavy metals bioconcentrations vary between fish species, sardines bioconcentrate low quantities of Pb, Cd, and Hg while swordfish has an intermediate concentration of Pb and Cd, but a low concentration of Hg [1], this concord with our results.

In sardine, Hg contamination level was slightly higher than in swordfish in our study, this can be explained by the fact that sardine is a species that lives near the coasts where marine pollution is very high [13]. In sardine (*S. pilchardus*), the Cd contamination average in our study was higher than four regions of the Atlantic coast of Morocco [14] but lower than reported in Saudi Arabia [15]. The Pb contamination average recorded in our study was higher than those reported by different authors, El Morhit *et al*. [16] (0.05 mg/kg), Olmedo *et al*. [1] (0.004 mg/kg), and Yabanli [17] (0.14 mg/kg). It remains much lower than the results reported by Amani *et al*. [15] (49 mg/kg). In the Atlantic Ocean (Morocco) and Turkey, the Hg contamination average registered on an average was higher than those reported by Chahid *et al*. [6] and Yabanli [17].

In swordfish (*Xiphias gladius*), the Cd concentration registered was higher than those reported by Zaza *et al*. [12], Jinadasa *et al*.[18] and, Kagi and Schaffer [19]. However, the Pb concentration recorded in our study in swordfish was higher than those documented by Zaza *et al*. [12] and by Jinadasa *et al*. [18]. Moualek [20] reported the following concentrations in western Algeria: 0.028, 0.038, 4.86, 3.20, and 6 mg/kg of fish weighing 13, 24, 32, 52, and 70 kg, respectively.

The Hg contamination average recorded in our study in the swordfish was lower than the concentration reported by Chikouche *et al*. [21] (0.96 mg/kg) and Jinadasa *et al*. [18] (1.24 and 0.9 mg/kg) in 2010 and 2013, respectively.

Several factors may be involved in explaining the differences between our results and those reported by other authors: Intrinsic factors: Fish size, age, sex, reproductive cycle, diet, and metabolic activity. The latter is proportional to heavy metals’ accumulation [22] and extrinsic factors: The environment whose fish live, which significantly affects the rate of contaminant accumulation by different organisms, as well as the concentrations of contaminants in the water column of fishing areas, handling, and processing fish during transportation and storage. The fishing season is also an important factor to consider as well as temperature, salinity, pH, and the presence of ligands in the marine environment [22]. Bioaccumulation also depends on the physical and chemical characteristics of the trace element concerned.

The Mediterranean Sea is shallow and almost completely closed; its full renewal takes more than a century by the Strait of Gibraltar (deep only 300 m). This is a real sea outfall of all types of waste and contaminant, as >500 million tons of sewage is discharged into every year, of which 120,000 tons of mineral oil, 6000 tons of Pb, and 3600 tons of phosphates [23].

Previous studies carried out on crustaceans and gastropods, considered as indicators of marine pollution, have shown that, on the Algerian east coast, Pb dominates with higher rates [24], while on the west coast, Cd reached 1.89 mg/kg [23].

These results indicate the levels of heavy metal pollution in these two fishing zones, which corroborate our results. In the sardine caught at El Tarf in the east, Ouldali *et al*. [25] reported a Hg concentration of 0.128 mg/kg.

The average concentrations of the three heavy metals in our study were quite high. Anthropogenic sources are numerous in the eastern region, as well as in central and west of the Algerian coast. These three selected fishing areas are the main coastal cities of the Mediterranean basin, but they lack effective purification systems. Where most of the industrial waste ends up, release detergents and other household and industrial chemicals into the sea; making the sea highly polluted. The city of Oran is the fourth polluting city on the Algerian west coast, discharging 140,000 m^3^/day of wastewater, 90% of them reach the sea directly [24]. The port areas of these three regions have a very important maritime activity related to the oil activity (Algiers, Oran) and commercial and fishing. Marine traffic releases very large quantities of pollutants, in particular, Pb (gasoline from ships, transport of petroleum products, paint from boats etc) and thus contributes to the sea water contamination [24]. The overall contamination averages and those reported by fishing regions reported in sardine exceed by far the thresholds set at 0.3, 0.1, and 0.5 mg/kg, respectively, for Pb, Cd, and Hg by both the Algerian [26] and European regulations [5].

Contamination of swordfish with Cd and Pb exceeded the thresholds set by the Algerian and European regulations, which is 0.3 mg/kg fresh weight for both metals. Hg’s concentrations (0.56 mg/kg), however, remained below the regulatory thresholds set at 1 mg/kg fresh weight.

These high values indicate the serious dangers to which the consumers are exposed, but this is a risk if the probability of exposure and the generalized risk factors for the population are calculated. Since toxicity is linked to the process of bioaccumulation, provisional tolerable weekly doses (PTWI) have been estimated for each metal by the European health authorities.

## Conclusion

Sardine and swordfish are contaminated with toxic heavy metals at very high levels which are exceeding the thresholds established by the Algerian and the European health authorities.

The sardine samples collected in different fishing areas show the variation of the contamination, related to the numerous sources of contamination whether natural or especially anthropic. Sardine is the most consumed fish in Algeria due to its low market price and availability, the average contamination, we reported the serious potential danger it poses to the consumer. A subsequent study of the probability of exposure will strengthen the estimate of the health risk incurred. The exhaustive and periodic control of heavy metals in fish caught locally is not mandatory, the Algerian health authorities, in terms of public health, must be concern about the toxic dangers of heavy metals in sardine and swordfish, and the risks consumer is exposed to.

## Authors’ Contributions

FM planned the study and drafted the manuscript under supervision of LB. FM and LB designed the experimental protocol. FM collected and analyzed the samples. FR participated in the analysis of the samples. FM did the statistical analysis. FM, LB, OA, and AHH corrected the manuscript. All authors read and approved the final manuscript.
